# AGING ON THE JOB? THE ASSOCIATION BETWEEN OCCUPATIONAL CHARACTERISTICS AND ACCELERATED BIOLOGICAL AGING

**DOI:** 10.1093/geroni/igac059.2497

**Published:** 2022-12-20

**Authors:** Theresa Andrasfay, Jung Ki Kim, Jennifer Ailshire, Eileen Crimmins

**Affiliations:** University of Southern California, Leonard Davis School of Gerontology, Los Angeles, California, United States; University of Southern California, Los Angeles, California, United States; University of Southern California, Los Angeles, California, United States; University of Southern California, Los Angeles, California, United States

## Abstract

There is a common saying that demanding jobs can make workers age faster, but there is little empirical evidence linking occupational characteristics to accelerated biological aging. We examine how occupational category (professional/managerial, sales/clerical, service, and manual) and occupational characteristics (e.g., psychosocial stressors, physical demands) are associated with a novel measure of physiologic aging, expanded biological age, that incorporates 22 biomarkers and captures physiologic dysregulation throughout several bodily systems. We assess how occupational characteristics for working individuals aged 51-60 in 2010 Health and Retirement Study (HRS) are associated with expanded biological age in the 2016 Venous Blood Study (VBS). We find that, compared to same-age individuals working in professional or managerial positions, those working in manual occupations appear 0.96-years older biologically while those in service jobs appear 2.8-years older biologically. Individuals whose jobs are high-stress, physically demanding, or require long working hours are nearly one year older biologically than their same-age peers without these adverse working conditions. These findings largely persisted after adjustment for educational attainment. Together these findings suggest that occupational characteristics may be an independent social determinant of accelerated aging.

